# Effectiveness of N95 Respirator Decontamination and Reuse against SARS-CoV-2 Virus

**DOI:** 10.3201/eid2609.201524

**Published:** 2020-09

**Authors:** Robert J. Fischer, Dylan H. Morris, Neeltje van Doremalen, Shanda Sarchette, M. Jeremiah Matson, Trenton Bushmaker, Claude Kwe Yinda, Stephanie N. Seifert, Amandine Gamble, Brandi N. Williamson, Seth D. Judson, Emmie de Wit, James O. Lloyd-Smith, Vincent J. Munster

**Affiliations:** National Institute of Allergy and Infectious Diseases, Hamilton, Montana, USA (R.J. Fischer, N. van Doremalen, S. Sarchette, M.J. Matson, T. Bushmaker, C.K. Yinda, S.N. Seifert. B.N. Williamson, E. de Wit, V.J. Munster);; Princeton University, Princeton, New Jersey, USA (D.H. Morris);; Marshall University, Huntington, West Virginia, USA (M.J. Matson);; University of California, Los Angeles, Los Angeles, California, USA (A. Gamble, J.O. Lloyd-Smith);; University of Washington, Seattle, Washington, USA (S.D. Judson)

**Keywords:** COVID-19, SARS-CoV-2, severe acute respiratory syndrome coronavirus 2, viruses, respiratory infections, zoonoses, coronavirus disease, N95 respirator, decontamination

## Abstract

The coronavirus pandemic has created worldwide shortages of N95 respirators. We analyzed 4 decontamination methods for effectiveness in deactivating severe acute respiratory syndrome coronavirus 2 virus and effect on respirator function. Our results indicate that N95 respirators can be decontaminated and reused, but the integrity of respirator fit and seal must be maintained.

The unprecedented pandemic of coronavirus disease has created worldwide shortages of personal protective equipment, in particular respiratory protection such as N95 respirators (*1*). Transmission of severe acute respiratory syndrome coronavirus 2 (SARS-CoV-2) occurs frequently in hospital settings; numerous reported cases of nosocomial transmission highlight the vulnerability of healthcare workers (*2*). The environmental stability of SARS-CoV-2 virus underscores the need for rapid and effective decontamination methods. 

In general, N95 respirators are designed for one use before disposal. Extensive literature is available for decontaminating N95 respirators of either bacterial spores, bacteria, or respiratory viruses (e.g. influenza A virus) (*3*–*6*). Effective inactivation methods for these pathogens and surrogates include UV light, ethylene oxide, vaporized hydrogen peroxide (VHP), gamma irradiation, ozone, and dry heat (A. Cramer et al., unpub data, https://doi.org/10.1101/2020.03.28.20043471) (*3*–*6*). The filtration efficiency and fit of N95 respirators has been less well explored, but reports suggest that both filtration efficiency and N95 respirator fit can be affected by the decontamination method used (*7*; Appendix).

We analyzed 4 different decontamination methods, UV light (260–285 nm), 70ºC dry heat, 70% ethanol, and VHP, for their ability to reduce contamination with infectious SARS-CoV-2 and their effect on N95 respirator function. The starting inoculum of SARS-CoV-2 has cycle threshold values of 20–22, similar to those observed in samples obtained from the upper and lower respiratory tract in humans. For each of the decontamination methods, we compared the normal inactivation rate of SARS-CoV-2 virus on N95 filter fabric to that on stainless steel. Using quantitative fit testing, we measured the filtration performance of N95 respirators after each decontamination run and 2 hours of wear, for 3 consecutive decontamination and wear sessions (Appendix). VHP and ethanol yielded extremely rapid inactivation both on N95 and on stainless steel (Figure, panel A). UV light inactivated SARS-CoV-2 virus rapidly from steel but more slowly on N95 fabric, probable because of its porous nature. Heat caused more rapid inactivation on N95 than on steel; inactivation rates on N95 were comparable to UV.

**Figure:**
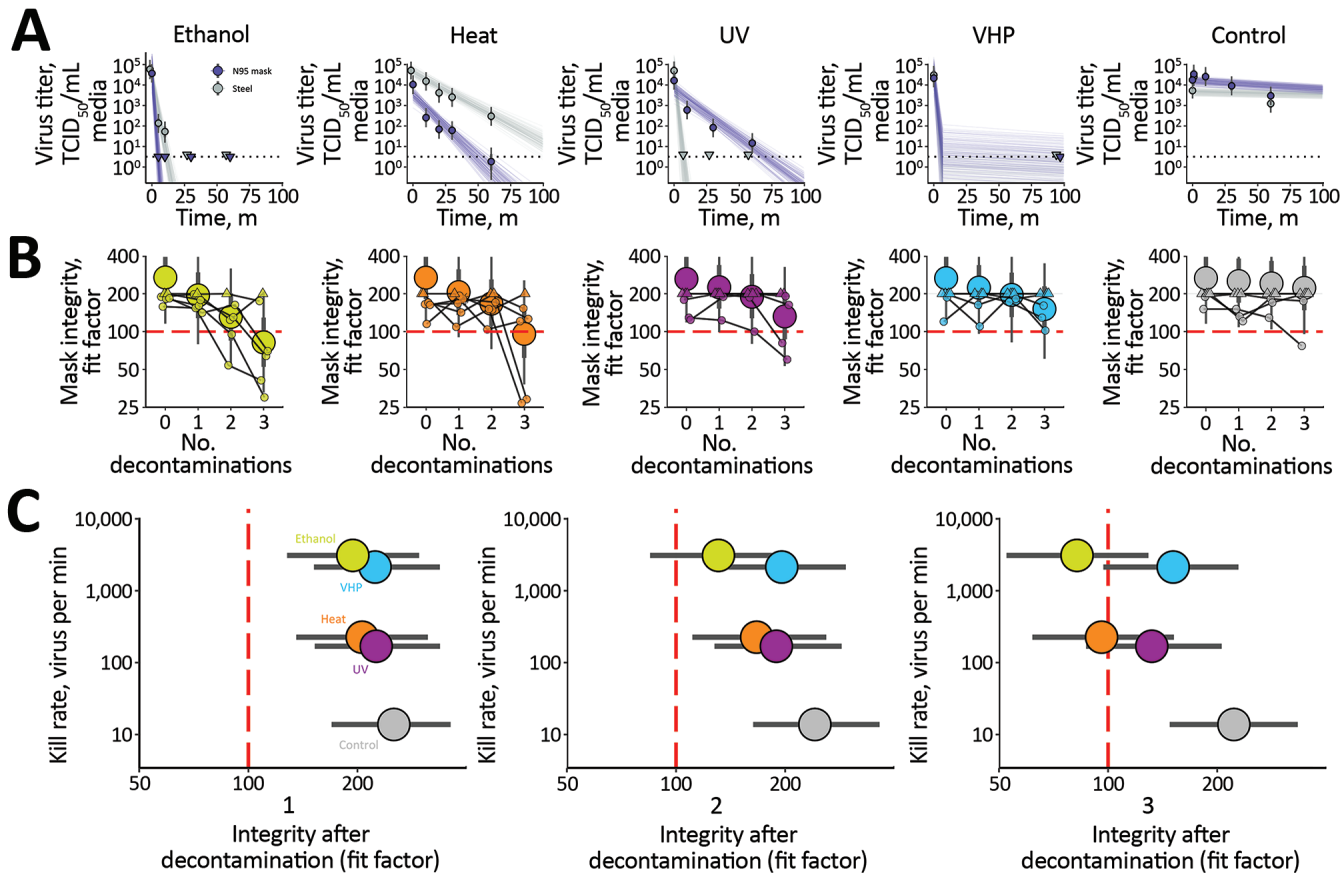
Results of decontamination of N95 respirators by 4 different methods. A) Inactivation of severe acute respiratory syndrome coronavirus 2 (SARS-CoV-2) virus (Appendix). Points indicate estimated mean viable titer across 3 replicates, circles the posterior median estimate of the mean, thick bars a 68% credible interval, and thin bars a 95% credible interval. Lines show predicted decay of virus titer over time and were generated by 50 random draws/replicate from the joint posterior distribution of the exponential decay rate (negative of the slope) and intercept (initial virus titer). Time points with no positive wells for any replicate are plotted as triangles at the approximate single-replicate LOD to indicate a plausible range of sub-LOD values. Black dotted line shows approximate LOD: 10^0.5^ TCID_50_/mL media. Points at the LOD and at t = 0 for ethanol and heat methods applied to steel are offset slightly up and to the left to avoid overplotting. B) Mask integrity quantitative fit testing results after decontamination and 2 hours of wear for 3 consecutive runs. Data from 6 individual replicates (small circles and triangles) for each treatment are shown, in addition to estimated median fit factor (large circles), 68% range of underlying fit factors (thick bars), and 95% range (thin bars). Fit factors are a measure of filtration performance, the ratio of the concentration of particles outside the mask to the concentration inside. The measurement machine reports values <200; measured values of 200 are shown as upward-pointing triangles to indicate that true underlying values may be higher; other measured values are shown as circles. A minimal fit factor of 100 (red dashed line) is required for a mask to pass a fit test. See also Appendix Figure 3. C) SARS-CoV-2 decontamination performance after 1, 2, and 3 decontamination cycles, shown as kill rate vs. mask integrity after decontamination. Circles represent estimated median, bar length estimated 68% range. LOD, limit of detection; TCID_50_, 50% tissue culture infective dose; VHP, vaporized hydrogen peroxide.

Quantitative fit tests showed that the filtration performance of the N95 respirator was not markedly reduced after a single decontamination for any of the 4 decontamination methods (Figure, panel B). Subsequent rounds of decontamination caused sharp drops in filtration performance of the ethanol-treated masks and, to a slightly lesser degree, the heat-treated masks. The VHP- and UV-treated masks retained comparable filtration performance to the control group after 2 rounds of decontamination and maintained acceptable performance after 3 rounds.

Our findings showed that VHP treatment had the best combination of rapid inactivation of SARS-CoV-2 virus and preservation of N95 respirator integrity under the experimental conditions (Figure, panel C). UV light killed the virus more slowly and preserved respirator function almost as well. Dry heat at 70ºC killed the virus with similar speed to UV and is likely to maintain acceptable fit scores for 1–2 rounds of decontamination but should not be used for 3 rounds. Consistent with earlier findings (*8*), ethanol decontamination reduced N95 integrity and is not recommended.

All treatments, particularly UV light and dry heat, should be conducted for long enough to ensure sufficient reduction in virus concentration. The degree of required reduction depends upon the degree of initial virus contamination. Policymakers can use our estimated decay rates together with estimates of real-world contamination to choose appropriate treatment durations (Appendix).

Our results indicate that, in times of shortage, N95 respirators can be decontaminated and reused up to 3 times by using UV light and HPV and 1–2 times by using dry heat. Following nationally established guidelines for fit testing, seal check, and respirator reuse is critical (*9**,**10*). We recommend performing decontamination for sufficient time and ensuring proper function of the respirators after decontamination using readily available qualitative fit testing tools. 

Appendix Additional information about N95 respirator decontamination and reuse against SARS-CoV-2 virus.
