# Supplementary material for: Effectiveness of N95 Respirator Decontamination and Reuse against SARS-CoV-2 Virus
Source: Emerg Infect Dis. 2020 Sep;26(9):2253–5. doi: 10.3201/eid2609.201524 (PMC7454118; doi:10.3201/eid2609.201524)
Supplement: Appendix — Additional information about N95 respirator decontamination and reuse against SARS-CoV-2 virus. [file 20-1524-Techapp-s1.pdf]

Article DOI: https://doi.org/10.3201/eid2609.201524

Effectiveness of N95 Respirator Decontamination and Reuse against SARS-CoV-2 Virus

Appendix

Methods

Literature Review on Mask Decontamination

The COVID-19 pandemic has highlighted the necessity for large-scale decontamination procedures for personal protective equipment (PPE), in particular N95 respirator masks (*1*). SARS-CoV-2 has frequently been detected on PPE of healthcare workers (*2*). The environmental stability of SARS-CoV-2 underscores the need for rapid and effective decontamination methods (*3*). Extensive literature is available for decontamination procedures for N95 respirators, using either bacterial spore inactivation tests, bacteria or respiratory viruses (e.g. influenza A virus) (*4–11*). Effective inactivation methods for these pathogens and surrogates include UV, ethylene oxide, vaporized hydrogen peroxide (VHP), gamma irradiation, ozone, and dry heat (*4,6,8,10–12*). The filtration efficiency and N95 respirator fit has typically been less well explored, but suggest that filtration efficiency, fluid shielding, and N95 respirator fit can be affected by the decontamination method used (*13*); Cramer et al., unpub. data, https://doi.org/10.1101/2020.03.28.20043471). It will therefore be critical to follow FDA, CDC, and OSHA guidelines in the United States, or appropriate local and national guidelines, for fit testing, seal check and respirator reuse (*4,14–17*).

Laboratory Experiments

Viruses and Titration

HCoV-19 nCoV-WA1-2020 (MN985325.1) was the SARS-CoV-2 strain used in our comparison (*18*). Virus was quantified by endpoint titration on Vero E6 cells as described previously (*19*). Cells were inoculated with 10-fold serial dilutions in 4-fold of samples taken from N95 mask and stainless steel surfaces as described below. One hour after inoculation of

cells, the inoculum was removed and replaced with 100 µL (virus titration) DMEM (Sigma-Aldrich) supplemented with 2% fetal bovine serum, 1 mM L-glutamine, 50 U/mL penicillin, and 50 µg/mL streptomycin. Six days after inoculation, cytopathogenic effect was scored and the $TCID_{50}$ was calculated (see below). Wells presenting cytopathogenic effects due to media toxicity (e.g., due to the presence of ethanol or hydrogen peroxide) rather than viral infection were removed from the titer inference procedure.

N95 and Stainless Steel Surface

N95 material discs were made by punching 9/16" (15 mm) fabric discs from N95 respirators, AOSafety N9504C respirators (Aearo Company, Southbridge, MA). The stainless steel 304 alloy discs were purchased from Metal Remnants (https://metalremnants.com) as described previously. 50 µL of SARS-CoV-2 was spotted onto each disc. A 0-timepoint measurement was taken before exposing the discs to the disinfection treatment. At each sampling timepoint, discs were rinsed 5 times by passing the medium over the stainless steel or through the N95 disc. The medium was transferred to a vial and frozen at -80°C until titration. All experimental conditions were performed in triplicate.

Decontamination Methods

Ultraviolet light. Plates with fabric and steel discs were placed under an LED high power UV germicidal lamp (effective UV wavelength 260–285 nm) without the titanium mesh plate (LEDi2, Houston, Tx) 50 cm from the UV source. At 50 cm the UVC power was measured by the manufacturer at 550 $\mu W/cm^2$. Plates were removed at 10, 30 and 60 minutes and 1 mL of cell culture medium added. The energy the discs were exposed to at 10, 30 and 60 min is 0.33 J/cm^2, 0.99 J/cm^2, and 1.98 J/cm^2 respectively. While the CDC has no specific recommendations on the minimum dose, they do report that a 1 J/cm2 dose can reduce tested viable viral loads by 99.9% (*4*).

Heat treatment. Plates with fabric and steel discs were placed in a 70°C oven. Plates were removed at 10, 20, 30, and 60 minutes and 1 mL of cell culture medium added.

70% ethanol. Fabric and steel discs were placed into the wells of one 24-well plate per timepoint and sprayed with 70% ethanol to saturation. The plate was tipped to near vertical and 5

passes of ethanol were sprayed onto the discs from ≈10 cm. After 10 minutes, 1 mL of cell culture medium was added.

VHP. Plates with fabric and steel discs were placed into a Panasonic MCO-19AIC-PT (PHC Corp., https://www.phchd.com) incubator with VHP generation capabilities and exposed to hydrogen peroxide (≈1,000 ppm). The exposure to VHP was 10 min; after the inactivation of the hydrogen peroxide, the plate was removed and 1 mL of cell culture medium was added.

Control. Plates with fabric and steel discs and steel plates were maintained at 21–23°C and 40% relative humidity for ≤4 days. After the designated timepoints, 1 mL of cell culture medium was added.

N95 Mask Integrity Testing

N95 mask (3M Aura Particulate Respirator 9211+/37193; https://www.3m.com) integrity testing after 2 hours of wear and decontamination, for 3 consecutive rounds, was performed for a total of 6 times for each decontamination condition and control condition. Masks were worn by subjects and integrity was quantitatively determined using the Portacount Respirator fit tester (838; TSI, https://tsi.com) with the N95 companion component, following the modified ambient aerosol condensation nuclei counter quantitative fit test protocol approved by the US Occupational Safety and Health Administration (*17*). Subjects were asked to bend over for 40 s, talk for 50 s, move head from side-to-side for 50 s, and move head up-and-down for 50 s while aerosols on inside and outside of mask were measured. By convention, this fit test is passed when the final score is ≥100. For N95 integrity testing, a Honeywell Mistmate humidifier (catalog #HUL520B; Honeywell, https://www.honeywell.com) was used for particle generation.

Statistical Analyses

In the model notation that follows, the symbol ~ denotes that a random variable is distributed according to the given distribution. Normal distributions are parametrized as Normal (mean, standard deviation). Positive-constrained normal distributions (“Half-Normal”) are parametrized as Half-Normal (mode, standard deviation). Normal distributions truncated to the interval [0, 1] are parameterized as TruncNormal (mode, standard deviation).

We use <Distribution Name>CDF (x | parameters) to denote the cumulative distribution function and and <Distribution Name>CCDF to denote complementary cumulative distribution

function of a probability distribution. So for example NormalCDF (5 | 0, 1) is the value of the Normal (0, 1) cumulative distribution function at 5.

We use logit (x) and invlogit (x) to denote the logit and and invlogit (x) to denote the inverse logit functions:

$$\text{logit}(x) = \ln \frac{x}{1 - x} \quad (1)$$

$$\text{invlogit}(x) = \frac{e^x}{1 + e^x} \quad (2)$$

Mean Titer Inference

We inferred mean titers across sets of replicates using a Bayesian model. The $\log_{10}$ titers v_{ijk} (the titer for the sample from replicate k of timepoint j of experiment i) were assumed to be normally distributed about a mean μ_{ij} with a standard deviation σ. We placed a very weakly informative normal prior on the $\log_{10}$ mean titers μ_{ij}:

$$\mu_{ij} \sim \text{Normal}\ (3, 5) \quad (3)$$

We placed a weakly informative normal prior on the standard deviation:

$$\sigma \sim \text{Normal}\ (0, 3) \quad (4)$$

We then modeled individual positive and negative wells for sample ijk according to a Poisson single-hit model (*20*). That is, the number of virions that successfully infect cells in a given well is Poisson distributed with mean:

$$V = \ln (2)\ 10^v \quad (5)$$

where v is the $\log_{10}$ virus titer in $TCID_{50}$, where v is the $\log_{10}$ virus titer in $TCID_{50}$, and the well is infected if at least one virion successfully infects a cell. The value of the mean derives from the fact that our units are $TCID_{50}$; the probability of infection at $v = 0$, i.e. 1 $TCID_{50}$, is equal to $1 - e^{-\ln (2) \times 1} = 0.5$.

Let Y_{ijkdl} be a binary variable indicating whether the lth well of dilution factor d (expressed as $\log_{10}$ dilution factor) of sample ijk was positive (so $Y_{ijkdl} = 1$ if the well was positive and 0 otherwise), which will occur as long as at least one virion successfully infects a cell.

It follows from (5) that the conditional probability of observing $Y_{ijkdl} = 1$ given a true underlying titer $\log_{10}$ titer v_{ijk} is given by:

$L\,(Y_{ijkdl} = 1 \mid v_{ijk}) = 1 - e^{-\ln(2) \times 10^{x}}$ (6)

Where

$x = v_{ijk} - d$ (7)

is the expected concentration, measured in $\log_{10}$ $TCID_{50}$, in the dilute sample. This is simply the probability that a Poisson random variable with mean $(-\ln(2) \times 10^{x}) > 0$. Similarly, the conditional probability of observing $Y_{ijkdl} = 0$ given a true underlying titer $\log_{10}$ titer v_{ijk} is given by:

$L\,(Y_{ijkdl} = 0 \mid v_{ijk}) = e^{-\ln(2) \times 10^{x}}$ (8)

which is the probability that the Poisson random variable is 0.

This gives us our likelihood function, assuming independence of outcomes across wells.

Virus Inactivation Regression

The durations of detectability depend on the decontamination treatment but also initial inoculum and sampling method, as expected. We therefore estimated the decay rates of viable virus titers using a Bayesian spline regression analogous to that used in van Doremalen et al. (*3*). This modeling approach allowed us to account for differences in initial inoculum levels across replicates as well as other sources of experimental noise. It also allows us to account for the fact that in the VHP experimental condition, viruses could not be sampled immediately following inactivation treatment. The model yields estimates of posterior distributions of viral decay rates and half-lives in the various experimental conditions – that is, estimates of the range of plausible values for these parameters given our data, with an estimate of the overall uncertainty (*21*).

Our data consist of 10 experimental conditions: 2 materials (N95 masks and stainless steel) by 5 treatments (no treatment, ethanol, heat, UV, and VHP). Each has 3 replicates, and multiple timepoints for each replicate. Treatments continue up to a time t^{treat}, but viruses may be sampled after t^{treat} in some conditions. We analyze the 2 materials separately. For each, we denote by Y_{ijkdl} the positive or negative status (see above) for well l which has dilution d for the titer v_{ijk} from experimental condition i during replicate j at timepoint k.

For each of the two materials, we model each replicate j for experimental condition i as starting with some true initial $\log_{10}$ titer $v_{ij}(0) = v_{ij0}$. We assume that viruses in experimental

condition i decay exponentially at a rate λ_i over time t for the duration of treatment, and at a rate λ_0 thereafter, where λ_0 is the exponential decay rate for material's control condition: no inactivation treatment. It follows that:

$v_{ij}(t) = v_{ij0} - \lambda_i t,\ t < t^{\text{treat}}$

$v_{ij}(t) = v_{ij0} - \lambda_i t^{\text{treat}} - \lambda_0 (t - t^{\text{treat}}),\ t > t^{\text{treat}}$ (9)

For all treatments except VHP, all sample $t \leq t^{\text{treat}}$, for VHP, $t^{\text{treat}} = 7$ min.

We use the direct-from-well data likelihood function described above, except that now instead of estimating titer distribution about a shared mean μ_{ij} we estimate λ_i under the assumptions that our observed well data Y_{ijkdl} reflect the titers $v_{ij}(t)$.

Regression Prior Distributions

We place a weakly informative Normal prior distribution on the initial $\log_{10}$ titers v_{ij0} to rule out implausibly large or small values (e.g. in this case undetectable $\log_{10}$ titers or $\log_{10}$ titers much higher than the deposited concentration), while allowing the data to determine estimates within plausible ranges:

$v_{ij0} \sim \text{Normal}(4.5, 3)$ (10)

We placed a weakly informative Half-Normal prior on the exponential decay rates λ_i:

$\lambda_i \sim \text{Half-Normal}(0, 20)$ (11)

Our plated samples were of volume 0.1 mL, so inferred titers were incremented by 1 to convert to units of $\log_{10}$ $TCID_{50}$/mL.

Mask Integrity Estimation

To quantify the decay of mask integrity after repeated decontamination, we used a logit-linear spline Bayesian regression to estimate the rate of degradation of mask fit factors over time, accounting for the fact that fit factors are interval-censored ratios. Fit factors are defined as the ratio of exterior concentration to interior concentration of a test aerosol. They are reported to the nearest integer, up to a maximum readout of 200, but arbitrarily large true fit factors are possible as the mask performance approaches perfect filtration.

We had 6 replicate masks j for each of 5 treatments i (no decontamination, ethanol, heat, UV, and VHP). Each mask j was assessed for fit factor at 4 timepoints k: before decontamination, and then after 1, 2, and 3 decontamination cycles. We label the control treatment $i = 0$. So we denote by F_{ijk} the fit factor for the jth mask from the ith treatment after k decontaminations (with $k = 0$ for the initial value).

We first converted fit factors F_{ijk} to the equivalent observed filtration rate Y_{ijk} by:

$Y = 1 - 1/F$ (12)

Observation Model and Likelihood Function

We modeled the censored observation process as follows. logit (Y_{ijk}) values are observed with Gaussian error about the true filtration logit (p_{ijk}), with an unknown standard deviation σ_o, and then converted to fit factors, which are then censored:

logit (Y_{ijk}) ~ Normal (logit (p_{ijk}), σ_o) (13)

Because our reported fit factors are known to be within integer values and right-censored at 200, for

$F_{ijk} \geq 200$ we have a conditional probability of observing the data given the parameters of

L (F_{ijk} | p_{ijk}, σ_o) = NormalCCDF (logit (1 – 1/200) | logit (p_{ijk}) σ_o) (14)

That is, we calculate the probability of observing a value of F greater than or equal to 200 (equivalent a value of Y greater than or equal to 1 – 1/200), given our parameters.

For $1.5 \leq F_{ijk} < 200$, we first calculate the upper and lower bounds of our observation $Y^{+}_{ijk} = 1 - 1 / (F_{\mathrm{ijk}} - 0.5)$ and $Y^{-}_{ijk} = 1 - 1 / (F_{ijk} - 0.5)$. Then:

L (F_{ijk} | p_{ijk}, σ_o) = NormalCDF (logit (Y^{+}_{ijk}) | logit (p_{ijk}) σ_o) –
NormalCDF (logit (Y^{-}_{ijk}) | logit (p_{ijk}) σ_o) (15)

That is, we calculate the probability of observing a value between Y^{+}_{ijk} and Y^{-}_{ijk}, given our parameters. In some cases, the difference $Y^{+}_{ijk} - Y^{-}_{ijk}$ may be less than the numerical precision of the computer program. In that case, we calculate:

L (F_{ijk} | p_{ijk}, σ_o) = NormalPDF (logit (Y^{+}_{ijk}) | logit (p_{ijk}) σ_o) (16)

Decay Model

We assumed that each mask had some true initial filtration rate p_{ij0}. We assumed that these were logit-normally distributed about some unknown mean mask initial filtration rate p_{avg} with a standard deviation σ_p, that is:

logit (p_{ij0}) ~ Normal (logit (p_{avg}), σ_p) (17)

We then assumed that the logit of the filtration rate, logit (p_{ijk}), after decontamination k was equal to logit ($p_{ij\,(k-1)}$) – d_{ijk}, where $d_{ijk} \geq 0$ is the degrading effect of the kth decontamination of treatment i on mask j. We assumed that the d_{ijk} were log-normally distributed about a treatment and decontamination-specific mean value μ_{dik} and decontamination round-specific standard deviation σ_{dik}:

log (d_{ijk}) ~ Normal (log (μ_{dik}), σ_{dik}) (18)

We defined the μ_{dik} as

$\mu_{dik} = \mu_{d0k} + \delta_{ik}$ (19)

where $\mu_{0k} \geq 0$ is natural degradation during the kth trial in the absence of decontamination (i.e. the degradation rate in the control treatment, $i = 0$), and $\delta_{ik} \geq 0$ is the additional mean effect of the kth decontamination treatment of type i, and δ_{0k} is set equal to 0.

Model Prior Distributions

We placed a weakly informative Half-Normal prior on the control mean degradation values μ_{0k} with a mode at zero (since it is possible that control masks decay minimally):

μ_{0k} ~ Half-Normal (0, 0.33) (20)

We placed a weakly informative Half-Normal prior on the non-control mean degradation values μ_{ik}, $i > 0$:

μ_{ik} ~ Half-Normal (0.25, 0.5) (21)

reflecting the conservative assumption that decontamination should degrade the mask at least somewhat.

We placed a Truncated Normal prior on the mean initial filtration p_{avg}:

p_{avg} ~ TruncNormal (0.99, 0.0025) (22)

The mode of 0.995 corresponds to the maximum measurable fit factor of 200. The standard deviation of 0.0025 leaves it plausible that some masks could start near or below the minimum acceptable threshold fit factor of 100, which corresponds to a p of 0.99.

We placed weakly informative Half-Normal priors on the logit-space standard deviations σ_p and σ_o. σ_p reflects variation in individual masks' initial filtration about p_{avg} and σ_o reflects noise in the observation process.

σ_p, σ_o ~ Half-Normal (0, 0.5) (23)

σ_{dik}, and the various σ_{dik} reflect variation in masks' true degree of degradation between decontaminations about the expected degree of decay,

σ_{dik} ~ Half-Normal (0, log (1.5) / 3) (24)

We chose standard of 0.5 for σ_p and σ_o because a standard deviation of 1.5 (i.e. 3 σ in the hyperprior) in logit space corresponds to probability values being uniformly distributed between 0 and 1; we therefore wish to tell our model not to use larger values of σ_p, σ_o as these would squash all p_{ijk} to one of two modes, one at 0 and one at 1 (*22*). For the same reason, we chose log (1.5) / 3 for the prior standard deviation σ_{dik}, since it is the standard deviation of a log of a logit-space value.

Markov Chain Monte Carlo Methods

For all Bayesian models, we drew posterior samples using Stan (Stan Core Team 2018; https://mc-stan.org), which implements a No-U-Turn Sampler (a form of Markov Chain Monte Carlo), via its R interface RStan. We ran 4 replicate chains from random initial conditions for 2,000 iterations, with the first 1,000 iterations as a warmup/adaptation period. We saved the final 1,000 iterations from each chain, giving us a total of 4,000 posterior samples. We assessed convergence by inspecting trace plots and examining $\hat{R}$ and effective sample size (n_{eff}) statistics. For the mask fit model, we assessed appropriateness of prior distributions with prior predictive checks (Appendix Figure 4) and assessed goodness-of-fit with posterior predictive checks (Appendix Figure 5). For the decay rate model, we assessed goodness of fit by plotting model estimated decay against estimated titers (Figure 1; Appendix Figure 1, 2).

Limit of Detection (LOD)

Endpoint titration has an approximate limit of detection set by the volume of the undilute sample deposited in each well. If all wells – including those containing undiluted sample – are negative and a Poisson single-hit model is used, the best guess is that the true titer lies somewhere below 1 $TCID_{50}$ / (volume of deposited sample). How far below is determined by the number of wells. For 4 wells, as was standard in our experiments, the first quarter log_{10} titer at which 0 wells is the most likely outcome is $10^{-0.5}$ $TCID_{50}$ per volume of sample. This is also the imputed Speaman-Karber titer in that case. Since we used samples of volume 0.1 mL, this corresponds to a value of $10^{0.5}$ $TCID_{50}$/mL. So although we do not use the Spearman-Karber method here (since we infer mean titers directly from the well data) we use that LOD value to plot samples for which no replicate had a positive well (since the posterior distribution in that case covers a wide-range of sub-threshold values).

Code and Data Availability

Code and data to reproduce the Bayesian estimation results and produce corresponding figures are archived online at OSF: https://doi.org/10.17605/OSF.IO/mkg9b and available on Github: https://github.com/dylanhmorris/n95-decontamination

References

1. Ranney ML, Griffeth V, Jha AK. Critical supply shortages—the need for ventilators and personal protective equipment during the Covid-19 pandemic. N Engl J Med. 2020;382:e41. PubMed https://doi.org/10.1056/NEJMp2006141

2. Ong SWX, Tan YK, Chia PY, Lee TH, Ng OT, Wong MSY, et al. Air, surface environmental, and personal protective equipment contamination by severe acute respiratory syndrome coronavirus 2 (SARS-CoV-2) from a symptomatic patient. JAMA. 2020;323:1610. PubMed https://doi.org/10.1001/jama.2020.3227

3. van Doremalen N, Bushmaker T, Morris DH, Holbrook MG, Gamble A, Williamson BN, et al. Aerosol and surface stability of SARS-CoV-2 as compared with SARS-CoV-1. N Engl J Med. 2020;382:1564–7. PubMed https://doi.org/10.1056/NEJMc2004973

4. US Centers for Disease Control and Prevention. Decontamination and reuse of filtering facepiece respirators. 2020 [cited 2020 Apr 5]. https://www.cdc.gov/coronavirus/2019-ncov/hcp/ppe-strategy/decontamination-reuse-respirators.html

5. US Food and Drug Administration. Final report for the Bioquell hydrogen peroxide vapor (HPV) decontamination for reuse of N95 respirators. 2016 [cited 2020 Apr 11]. https://www.fda.gov/media/136386/download

6. Fisher EM, Shaffer RE. A method to determine the available UV-C dose for the decontamination of filtering facepiece respirators. J Appl Microbiol. 2011;110:287–95. PubMed https://doi.org/10.1111/j.1365-2672.2010.04881.x

7. Heimbuch BK, Kinney K, Lumley AE, Harnish DA, Bergman M, Wander JD. Cleaning of filtering facepiece respirators contaminated with mucin and *Staphylococcus aureus*. Am J Infect Control. 2014;42:265–70. PubMed https://doi.org/10.1016/j.ajic.2013.09.014

8. Heimbuch BK, Wallace WH, Kinney K, Lumley AE, Wu CY, Woo MH, et al. A pandemic influenza preparedness study: use of energetic methods to decontaminate filtering facepiece respirators contaminated with H1N1 aerosols and droplets. Am J Infect Control. 2011;39:e1–9. PubMed https://doi.org/10.1016/j.ajic.2010.07.004

9. Lin TH, Tang FC, Hung PC, Hua ZC, Lai CY. Relative survival of *Bacillus subtilis* spores loaded on filtering facepiece respirators after five decontamination methods. Indoor Air. 2018;28:754–62. PubMed https://doi.org/10.1111/ina.12475

10. Mills D, Harnish DA, Lawrence C, Sandoval-Powers M, Heimbuch BK. Ultraviolet germicidal irradiation of influenza-contaminated N95 filtering facepiece respirators. Am J Infect Control. 2018;46:e49–55. PubMed https://doi.org/10.1016/j.ajic.2018.02.018

11. Viscusi DJ, Bergman MS, Eimer BC, Shaffer RE. Evaluation of five decontamination methods for filtering facepiece respirators. Ann Occup Hyg. 2009;53:815–27. PubMed

12. US Centers for Disease Control and Prevention. Chemical disinfectants: guideline for disinfection and sterilization in healthcare facilities. 2008 [cited 2020 Apr 11]. https://www.cdc.gov/infectioncontrol/guidelines/disinfection/disinfection-methods/chemical.html#Hydrogen

13. Lin TH, Chen CC, Huang SH, Kuo CW, Lai CY, Lin WY. Filter quality of electret masks in filtering 14.6-594 nm aerosol particles: effects of five decontamination methods. PLoS One. 2017;12:e0186217. PubMed https://doi.org/10.1371/journal.pone.0186217

14. US Food and Drug Administration. N95 respirators and surgical masks (face masks). 2020 [cited 2020 Apr 5]. https://www.fda.gov/medical-devices/personal-protective-equipment-infection-control/n95-respirators-and-surgical-masks-face-masks

15. US Occupational Safety and Health Administration. Temporary enforcement guidance—healthcare respiratory protection annual fit-testing for N95 filtering facepieces during the COVID-19 outbreak. 2020 [cited 2020 Apr 11]. https://www.osha.gov/memos/2020-03-14/temporary-enforcement-guidance-healthcare-respiratory-protection-annual-fit

16. US Occupational Safety and Health Administration. User seal check procedures (mandatory). 2020 [cited 2020 Apr 11] https://www.osha.gov/laws-regs/regulations/standardnumber/1910/1910.134AppB1

17. US Occupational Safety and Health Administration. Transcript for the OSHA training video entitled respirator fit testing. 2012 [cited 2020 Apr 10]. https://www.osha.gov/video/respiratory_protection/fittesting_transcript.html

18. Holshue ML, DeBolt C, Lindquist S, Lofy KH, Wiesman J, Bruce H, et al.; Washington State 2019-nCoV Case Investigation Team. First case of 2019 novel coronavirus in the United States. N Engl J Med. 2020;382:929–36. PubMed https://doi.org/10.1056/NEJMoa2001191

19. van Doremalen N, Bushmaker T, Munster VJ. Stability of Middle East respiratory syndrome coronavirus (MERS-CoV) under different environmental conditions. Euro Surveill. 2013;18:20590. PubMed https://doi.org/10.2807/1560-7917.ES2013.18.38.20590

20. Brownie C, Statt J, Bauman P, Buczynski G, Skjolaas K, Lee D, et al. Estimating viral titres in solutions with low viral loads. Biologicals. 2011;39:224–30. PubMed https://doi.org/10.1016/j.biologicals.2011.06.007

21. Gelman A, Carlin J, Stern H, Dunson D, Vehtari A, Rubin D. Bayesian data analysis. 3rd edition. Boca Raton: CRC Press; 2014.

22. Northrup JM, Gerber BD. A comment on priors for Bayesian occupancy models. PLoS One. 2018;13:e0192819. PubMed https://doi.org/10.1371/journal.pone.0192819

Appendix Table. Effect of decontamination method on SARS-CoV-2 viability on N95 respirators

		Half-life, min			Time to one thousandth, min			Time to one millionth, min		
Treatment	Material	Median	2.5%	97.5%	Median	2.5%	97.5%	Median	2.5%	97.5%
Control	N95 mask	78.5	66	90.2	782	658	899	1.56×10^3	1.32×10^3	1.8×10^3
	Steel	234	202	260	2.33×10^3	2.01×10^3	2.59×10^3	4.66×10^3	4.02×10^3	5.18×10^3
Ethanol	N95 mask	0.35	0.257	0.42	3.49	2.56	4.19	6.97	5.11	8.37
	Steel	0.89	0.729	1.04	8.87	7.27	10.4	17.7	14.5	20.8
Heat	N95 mask	4.8	3.96	5.62	47.8	39.4	56	95.6	78.9	112
	Steel	8.85	7.45	10.2	88.2	74.2	102	176	148	204
UV	N95 mask	6.41	5.45	7.3	63.8	54.3	72.8	128	109	146
	Steel	0.491	0.31	0.612	4.89	3.09	6.1	9.79	6.18	12.2
VHP	N95 mask	0.508	0.312	0.67	5.06	3.11	6.67	10.1	6.22	13.3
	Steel	0.429	0.287	0.533	4.28	2.86	5.31	8.55	5.72	10.6

*Results are reported as the median and upper- and lower-limits of the 95% credible interval of the estimated half-life, and time needed to reduce viable SARS-CoV-2 load by a factor of 1,000 or 1,000,000, based on the posterior distribution of the exponential decay rate of the virus on different materials following different decontamination treatments.

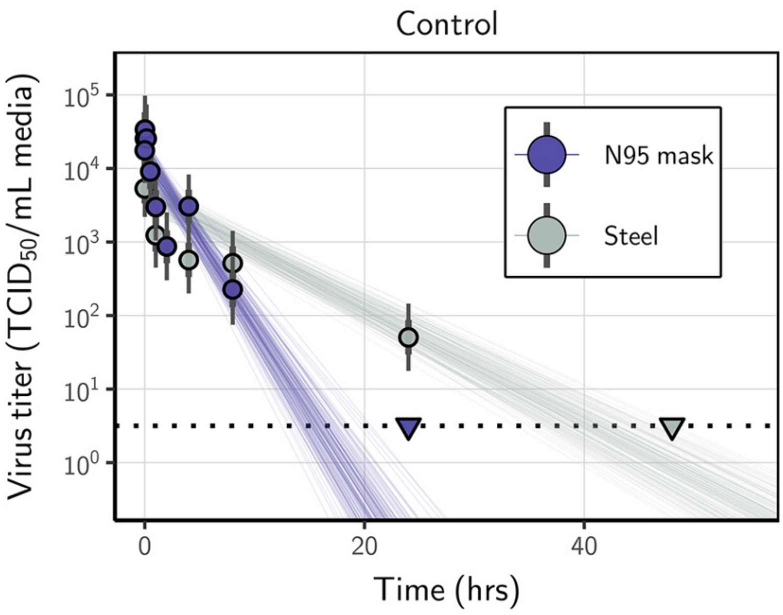

Appendix Figure 1. Exponential decay fits for control experiments, carried out to final timepoints (a limited subset are shown in Figure 1 of the main text to make measurements for treatments readable). Lines show predicted decay of virus titer over time (lines; 50 random draws per replicate from the joint posterior distribution of the exponential decay rate, i.e. negative of the slope, and intercept, i.e. initial virus titer). Points show estimated mean titer across three replicates (circles, thick bars, and thin bars show the posterior median estimate of this mean, a 68% credible interval, and a 95% credible interval, respectively). Timepoints with no positive wells for any replicate are plotted as triangles at the approximate single-replicate detection limit of the assay (LOD, see Materials and Methods for discussion) to indicate that a range of sub-LOD values are plausible. Black dotted line shows approximate LOD: $10^{0.5}$ $TCID_{50}$/mL media.

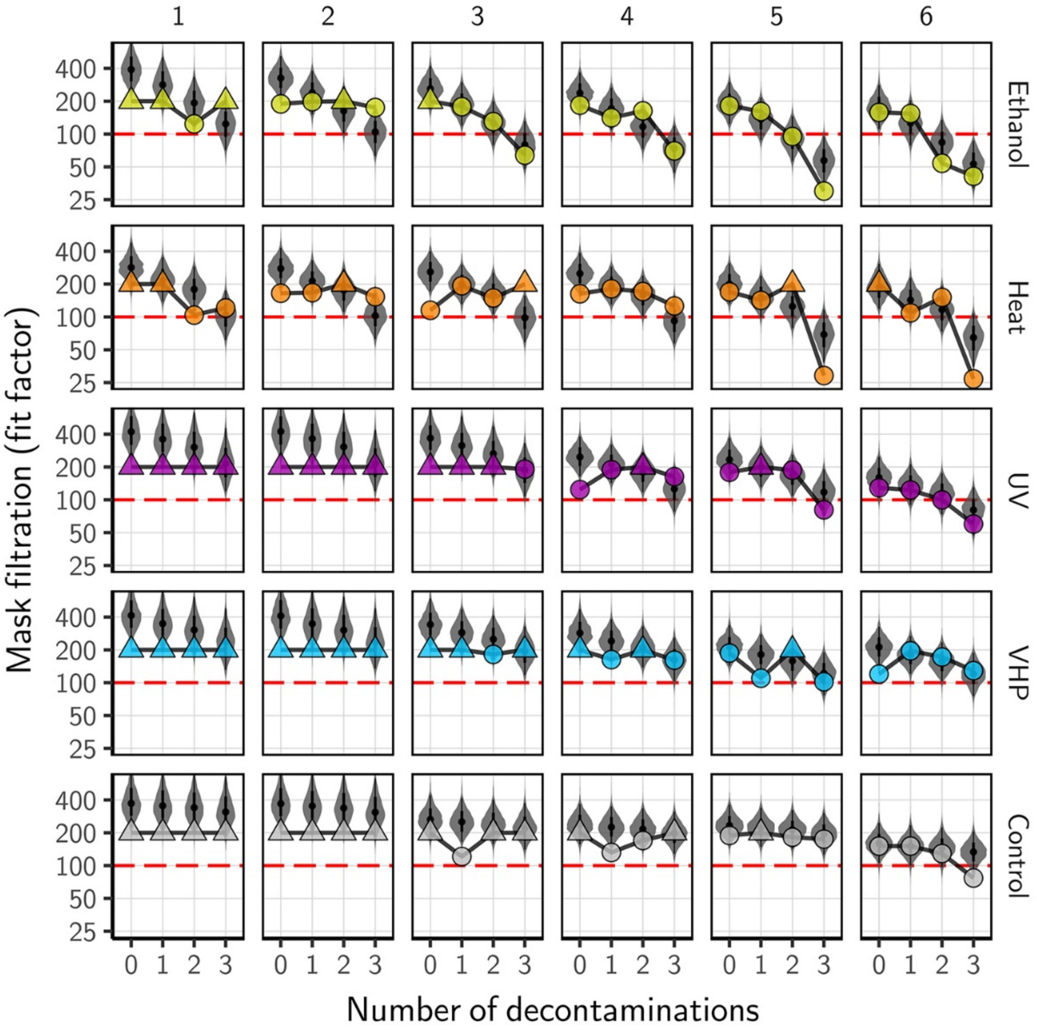

Appendix Figure 2. Estimated mask integrity over time for individual masks. Connected points represent observed values; violin plots show posterior distribution of estimated underlying integrity, measured as a fit factor. Black dots show posterior median estimates; thick bars, 68% credible intervals; and thin bars 95% credible intervals. Triangles denote observed values of 200, the upper limit of measurement (so true values may be higher). Circles denote other observed values. Red dashed line indicates fit factor of 100, the minimum passing value for a fit test. Columns are ordered by estimated mask integrity after the third decontamination.

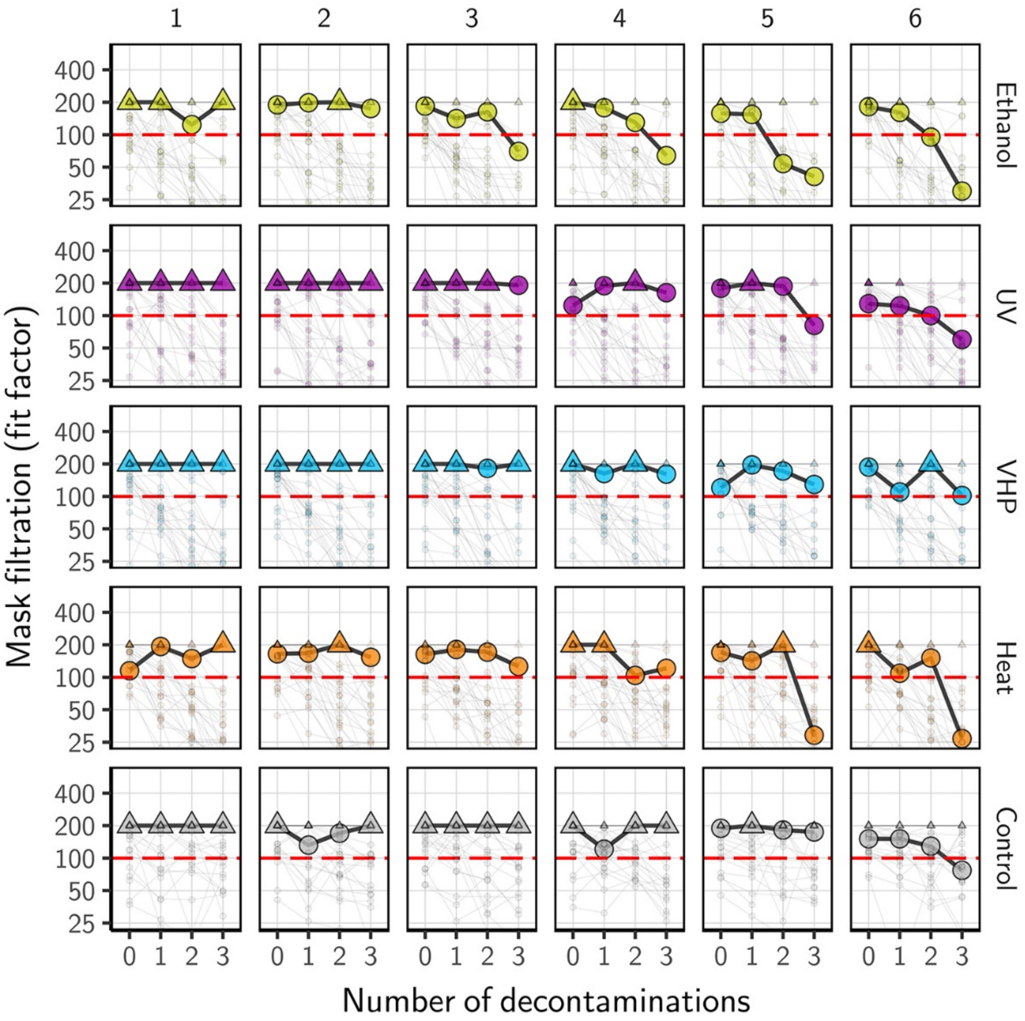

Appendix Figure 3. Prior predictive checks for mask fit model. Large connected points are observed values for individual masks. Small overplotted connected points are simulated observations drawn from the prior predictive distribution (including simulated measurement error and censoring). The wide range of simulated values, which covers the real data, shows that the priors are not overly informative. Triangles denote observed values of 200, the upper limit of measurement (so true values may be higher). Circles denote other observed values. Red dashed line indicates fit factor of 100, the minimum passing value for a fit test. Each panel shows 25 randomly chosen simulated observations. Masks are ordered by fit factor observed after 3 decontaminations.

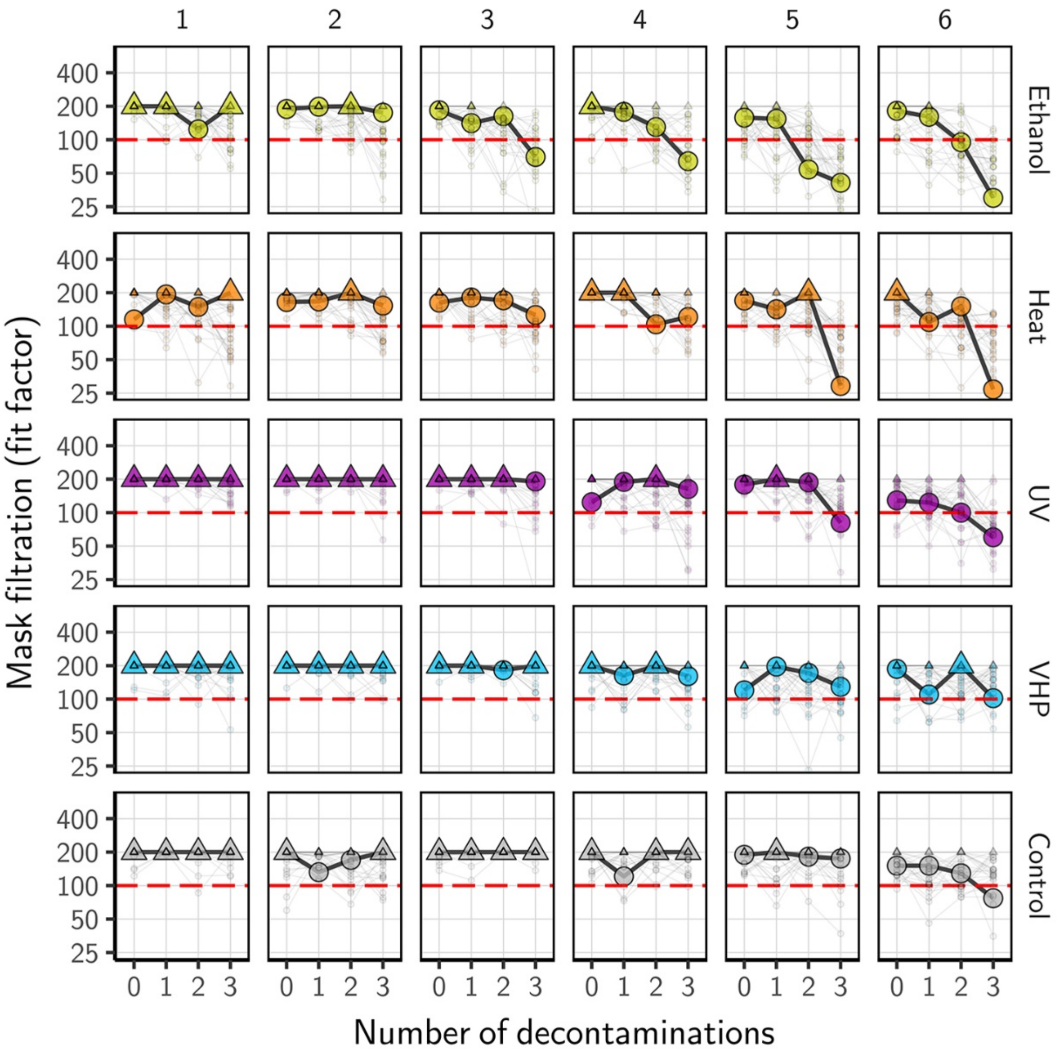

Appendix Figure 4. Posterior predictive checks for mask fit model. Large connected points are observed values for individual masks. Small overplotted connected points are simulated sets of observations for each mask, drawing from the posterior predictive distribution for that mask (including simulated measurement error and censoring). The relatively tight coverage of the actual observations suggests that the model fits the data well. Triangles denote observed values of 200, the upper limit of measurement (so true values may be higher). Circles denote other observed values. Red dashed line indicates fit factor of 100, the minimum passing value for a fit test. Each panel shows 25 randomly chosen simulated observations per panel. Masks are ordered by fit factor observed after 3 decontaminations.
